# Falls Among Residents Living in Care Homes Using Real‐Time Data Collection: A Large UK Case‐Control Study

**DOI:** 10.1002/hsr2.72350

**Published:** 2026-04-19

**Authors:** Julian Gertner, Ofir Dvir, Niv Shifrin, Paul Wolfson, Robert Moskovitch, Laurence B. Lovat

**Affiliations:** ^1^ Division of Surgery and Interventional Science University College London London UK; ^2^ Software and Information Systems Engineering Ben Gurion University of the Negev Beer Sheva Israel

**Keywords:** care home residents, care homes, digital health, fall risk, falls, observational study, real‐time data

## Abstract

**Background and Aims:**

Falls are common among older residents living in care homes and have significant associated morbidity and mortality. These incidents negatively impact quality of life and pose a substantial financial burden on healthcare systems.

**Methods:**

This case‐control study explored fall patterns and identifiable risk factors for falls using real‐time data collected via the Mobile Care Monitoring (MCM) app across 1,700 care homes in England. Care home staff continuously recorded data for 1 year on a matched cohort of 6,006 residents who experienced at least one fall and 6,006 who had no documented falls, aged 75 years and older.

**Results:**

The fall incidence rate was 1,249 per 1,000 residents per year and among those who had already experienced at least one fall, 45% had three or more falls. Advancing age was significantly associated with increasing fall risk (Incidence Rate Ratio (IRR): 1.11; 95% CI: 1.04–1.17; *p* < 0.001). Residents who experienced a fall were significantly more likely to have at least one hospitalisation than those who had no documented falls (54% vs. 34% respectively, *p* < 0.001). Falls with injury were significantly higher for males than females (IRR 1.79; 95% CI: 1.44–2.14; *p* < 0.001). 98.5% of falls occurred indoors, mostly in the bedroom, lounge, and bathroom. Logistic regression analysis identified nine variables significantly and independently associated with fall risk, including exercise frequency and fluid intake. There was no significant relationship between fall rates or hospitalisation rates, and Care Quality Commission (CQC) ratings of the care homes.

**Conclusion:**

The use of real‐time electronic data collection through the MCM app offers a robust foundation for addressing the challenges associated with falls in this population.

## Summary

- Continuous app‐based data collection across 1,700 care homes identified nine variables independently associated with increased fall risk, including low fluid intake and increased frequency of exercise and toileting.
- Nearly all falls occurred indoors, most commonly in bedrooms, lounges, and bathrooms.
- Falls were significantly associated with hospitalisations, but care home quality ratings (CQC) were not linked to fall or hospitalisation rates.

## Why Does This Paper Matter?

This is the largest study to use real‐time electronic data collection to investigate fall risk among residents living in care homes. It highlights the potential for identifying high‐risk residents living in care homes to guide targeted fall prevention strategies.

## 1 Introduction

According to the World Health Organization, a third of people aged over 70 fall and the likelihood rises with increasing age and frailty. Falls account for most injury‐related hospital admissions and 40% of injury‐related deaths in older persons [1]. In addition to the human cost of distress, pain, fractures and loss of independence [2], falls pose a substantial financial burden on healthcare systems with an estimated cost to the United Kingdom (UK) National Health Service (NHS) of £2.3 billion per year (approx. US$3.1 billion) [3]. Falls are common among residents living in care homes, with approximately 25% of falls causing serious injury. Furthermore, falls account for roughly 40% of hospital admissions originating from care homes [4, 5]. Over 80% of deaths from external causes among residents living in care homes are due to falls, and the incidence is rising with an ageing population [6]. In this study, the term ‘care home’ is used to encompass both residential and nursing homes to ensure consistency across the dataset, although specific cited literature may refer to these settings as ‘nursing homes’. UK care homes are staffed by care assistants, with registered nurses also present in facilities requiring 24 h a day nursing level care. Medical oversight is provided by General Practitioners (GPs) who are typically based off‐site and visit as needed.

Identifying the fall risk of residents living in care homes can facilitate targeted prevention [3]. Many fall risk assessment tools have been developed [7, 8]. The validated Missouri Alliance for Home Care Fall Risk Assessment Tool (MAHC‐10) shows good sensitivity (96.6%) but low specificity (13.3%) [9]. Despite multiple assessment tools, few algorithmic models have been developed to predict an individual's fall risk. All residents living in care homes are at high risk of falling, warranting a standard comprehensive assessment followed by multidomain interventions [7]. Nevertheless, identifying specific residents at highest risk remains an important challenge. We carried out this research to understand the patterns of falls in care homes by utilising unique routinely documented care home data from a mobile app to identify risk factors associated with falls that could provide the basis for future dynamic risk assessment tools.

## 2 Methods

### 2.1 Data Collection

Data were collected electronically from older residents living in care homes using the General Data Protection Regulation (GDPR)‐compliant Mobile Care Monitoring (MCM) mobile app (Person Centred Software, Guildford, UK). This digital care system is widely used in the UK. The mobile app enables carers to continuously record residents' activities and statuses, including food and fluid intake, medication administration, exercise activities, and mental stimulation. Happiness scores are recorded using a Likert scale with facial expressions depicting emotions such as smiley or sad faces [10]. Validated tools are commonly applied in care home settings for appetite [11], malnutrition [12], manual handling [13], and care dependency [14]. For fall risk assessment, various tools exist [7, 8, 9].

While the specific scales used within the MCM app were not available for analysis, data collection was managed by the app's uniform digital architecture. This system utilises a consistent set of icons and data‐entry fields across all participating care homes, providing a standardised format for data capture regardless of the internal protocols of an individual care home. Recordings are made continuously in real time using a simple, intuitive interface. The system is icon‐driven with limited need for typing and is suitable for non‐native English speakers, without any technical knowledge.

Staff members can document a resident drinking by selecting a picture of the resident followed by a glass of juice. The volume drunk is captured using a slider at the bottom of the screen, and the resident's mood using a smiley icon at the top.

Data are recorded by staff in real time rather than at the end of the shift and are automatically uploaded to a GDPR‐compliant central cloud‐based database providing a detailed record. This is available to carers, home managers and residents' families.

Secondary analytics were performed on a fully anonymised subset of the data from 2016 to 2018. Neither specific scoring systems for many assessments recorded within the MCM app nor data from free text fields were made available. Some care homes began using the MCM app during the study period. We determined the time taken for data collection protocols to stabilise in each care home. To reduce bias, we excluded data from care homes that began using the MCM app during the study period until their protocols had stabilised.

Falls were defined as events in which the person, unintentionally and regardless of cause, came to rest on the floor. Falls were reported on a specifically designed report form on the MCM app including person identification, date, time, location and severity of injury. Fall injury severity was judged by staff at the point of care and categorised using four available levels: major, minor, monitor, and no injury. These were subsequently grouped into a binary classification: falls with injury (major or minor injuries) and falls without injury (monitor or no injury). Classification details are provided in Supporting Table S3.

Ethical approval was obtained from the UCL Research Ethics Committee, Project ID/Title: 14223/001: CHART: Care Homes – Analysis of Digitally Recorded Data) in 2019. Completion of this work was significantly delayed by the coronavirus disease 2019 (COVID‐19) pandemic. As the study involved the secondary analysis of a fully anonymised dataset where participants had previously provided explicit consent for their data to be shared for research purposes, a further waiver of informed consent was not required.

### 2.2 Residents

**Figure 1:**
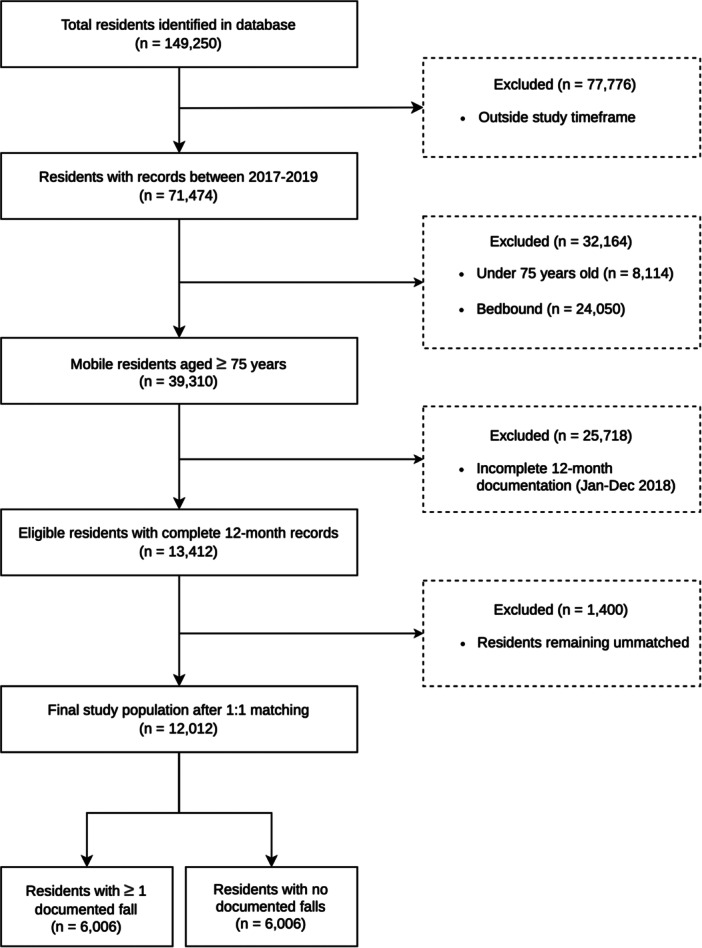
CONSORT flow diagram illustrating the selection process for residents living in care homes included in the analyses. Details include exclusion criteria for age, mobility status, and documentation duration.

Data were collected from over 1,700 care homes across England, from 2013 to 2019 in the MCM app. The total population included 149,250 residents, of whom 13,412 met the criteria of being aged 75 years or over, not bed‐bound, with at least one full year of MCM app documentation (Figure 1). Bed‐bound residents were defined as residents with documentation of ‘Turn’, ‘Reposition’ or ‘Assist to Reposition’.

This case‐control cohort study included two groups: residents who experienced at least one fall and those with no documented falls. Cases were residents with documentation of at least one fall with an injury of some level of severity during the year 2018. Controls were mobile residents with no documented falls, matched to cases based on their age and sex. Of the 13,412 residents meeting study criteria, 12,012 residents were matched: 6,006 who experienced at least one fall and 6,006 with no documented falls. The Care Quality Commission (CQC) is the independent regulator of health and social care in England and defines the care home quality using a four‐tier scale: Outstanding, Good, Requires Improvement, or Inadequate. An ‘Inadequate’ rating is the lowest classification, indicating the facility is performing significantly below expected standards and is subject to mandatory regulatory intervention to ensure that national standards of care are met [15]. We investigated types and patterns of falls, together with hospitalisation and CQC rating, and sought other recorded factors associated with falls. Hospitalisation data represents all‐cause admissions as the specific reasons for hospital admission were not captured within the app's data‐entry fields. Due to the fully anonymised nature of the dataset, linkage to external healthcare records to determine admission causes was not feasible.

### 2.3 Statistical Analysis

Descriptive data are presented as means, medians, numbers, percentages and incidence rates. To calculate fall incidence rates, the number of falls among individuals was divided by the individuals' aggregated exposure time. For comparison of fall incidence rates between subgroups, incidence rate ratios (IRR) with a 95% confidence interval (CI) were calculated. For comparison of fall incidence rates by age, the IRR represents the change for every increasing year.

To identify variables associated with the risk of a first fall, univariable and multivariable logistic regression analyses were performed. Data were taken from the 6 months preceding the first fall for residents who had experienced at least one fall, and from a random time point for residents with no documented falls. Data were aggregated per month and consisted of 34 routinely collected variables including weight, toileting frequency and fluid intake (see Supporting Table S1 for definitions). Odds Ratios (ORs) were calculated comparing residents who had experienced at least one fall vs. residents with no documented falls for each variable. Variables were first screened using univariable analysis; those demonstrating statistical significance (*p* < 0.05) were entered into a multivariable model to identify variables that remained independently associated with falling. All statistical tests were 2‐sided with an a priori significance level of α = 0.05. Statistical analyses were performed using R (version 4.1.0; R Foundation for Statistical Computing, Vienna, Austria) within the RStudio integrated development environment (version 1.4; RStudio, PBC, Boston, MA).

Fluid intake was scaled to a 1‐litre increment to represent a clinically relevant difference. To ensure the reliability of our results, the Events Per Variable (EPV) was calculated based on 6,006 events and 15 variables in the multivariable analysis. This equates to an EPV of 400, which exceeds the recommended thresholds for model stability.

A complete‐case analysis was performed, defined as residents with at least one full year of MCM app documentation. For activity‐based variables, non‐recorded events were treated as zero occurrences. To prioritise the identification of broad associations across over 1,700 care homes, a single‐level logistic regression was used rather than a clustered model. As the study aimed to identify independent associations rather than to validate a predictive algorithm, formal goodness‐of‐fit diagnostics were not performed. These tests are also known to be over‐sensitive in large‐scale datasets often yielding statistically significant results that do not reflect clinical model utility.

### 2.4 Declaration of Sources of Funding

This work was supported by a collaboration funded by the British Council Grant BIRAX [grant number 5531]. BIRAX played no role in the design, execution, analysis or interpretation of data, or writing of the study.

## 3 Results

### 3.1 Care Home Data Validity

**Figure 2:**
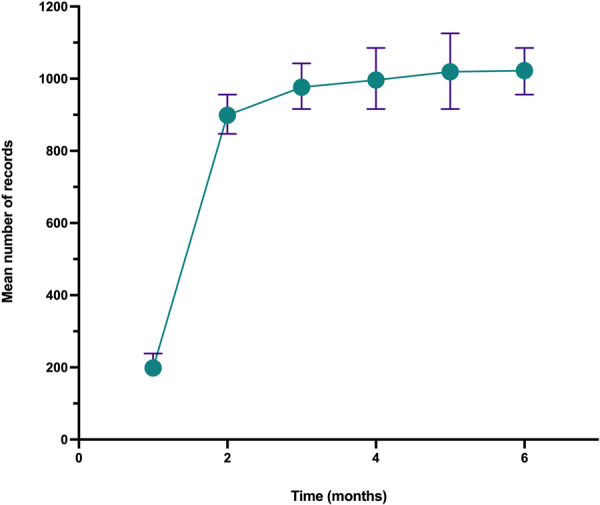
Mean records per resident per month during initial MCM app usage. Mean and Standard Deviations are shown for the number of digital care records collected per resident per month in the first six months of MCM app usage.

Care homes took 3 months to adjust to the new recording method using the MCM mobile app (Figure 2). The mean number of records collected per resident per month across all care homes stabilised at 1000 (approximately 32 records per resident per day). To ensure data integrity and allow staff time to learn the MCM app, data were taken only from care homes using the app by September 2017.

### 3.2 Falls Incidence and Characteristics

Data for 12,012 matched residents were included. 72% were female and the mean age (years ± SD) was 87.5 ± 5.8 for females and 85.7 ± 5.9 for males. There were 23,621 falls documented among the 6,006 residents who fell, 2,006 (33%) had one fall, 1,203 (20%) had two falls and 2,797 (47%) had at least three falls, ranging up to 23. The fall incidence rate was 1,249 (95% CI: 1,166–1,332) per 1,000 residents per year. There was no difference in the fall incidence rate between sexes (IRR for women compared to men: 1.23; 95% CI: 0.85–1.61; *p* = 0.79). However, advancing age was positively associated with a higher fall incidence rate (IRR: 1.11; 95% CI: 1.04–1.17; *p* < 0.001).

Documentation included an optional field for fall injury severity, which was completed for 15,199 (64.3%) of the documented falls. Among falls with documented injury severity, 9,950 (65%) resulted in no injury, 2,888 (19%) required monitoring, 1,984 (13.5%) led to minor injury and 377 (2.5%) sustained major injury. The falls incidence rate for falls with injury was significantly higher for males than females (IRR 1.79; 95% CI: 1.44–2.14; *p* < 0.001).

**Figure 3:**
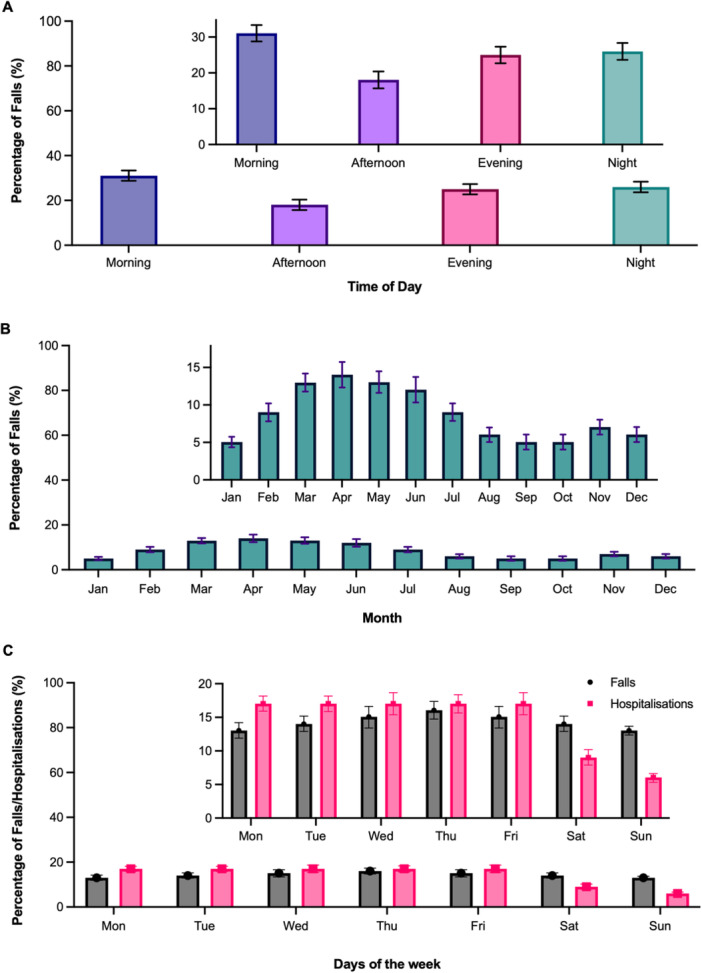
Proportion of falls and hospitalisations in residents living in care homes, by time of day, month, and day of the week. Panel A: Proportion of falls (%) by time of day, categorised as Morning (05:00–11:00), Afternoon (11:00–17:00), Evening (17:00–23:00), and Night (23:00–05:00). Differences between all time intervals were statistically significant (ANOVA *p* < 0.001). Panel B: Proportion of falls (%) across different months of the year. Panel C: Proportion of falls and hospitalisations (%) occurring on each day of the week. Error bars represent standard deviation. Panels A, B and C show the full scale, with inset panels displaying truncated y‐axes.

Falls occurred at the highest rate in the morning and at the lowest rate in the afternoon (Figure 3a). Differences were statistically significant between all time intervals (ANOVA *p* < 0.001 with differences between each time interval also *p* < 0.001 using Tukey's multiple comparisons test). The monthly fall incidence was the highest between February and June (Figure 3b).

Location was documented for 16,791 falls. 98.5% occurred indoors, with the bedroom (52%), lounge (15%) and the bathroom (13%) being the commonest indoor places.

### 3.3 Hospitalisation

There were 26,723 hospitalisations among 5,364 residents, representing 45% of the total study population. The overall hospitalisation incidence rate was 1375 (95% CI: 1270–1481) per 1,000 resident years. Residents who experienced a fall were significantly more likely to have at least one hospitalisation than those who had no documented falls (54% vs. 34% respectively, *p* < 0.001). While the fall incidence rate was not related to sex, the hospitalisation incidence rate was 13% lower in women (IRR for women compared to men: 0.87; 95% CI: 0.76–0.98; *p* < 0.001). Older residents were less likely to be hospitalised after a major fall compared with younger residents (IRR: 0.91; 95% CI: 0.83–0.99; *p* < 0.001).

Falls occurred at similar rates throughout the week, but there was a lower frequency of hospitalisations over the weekend (Sunday and Saturday) (Figure 3c).

**Figure 4:**
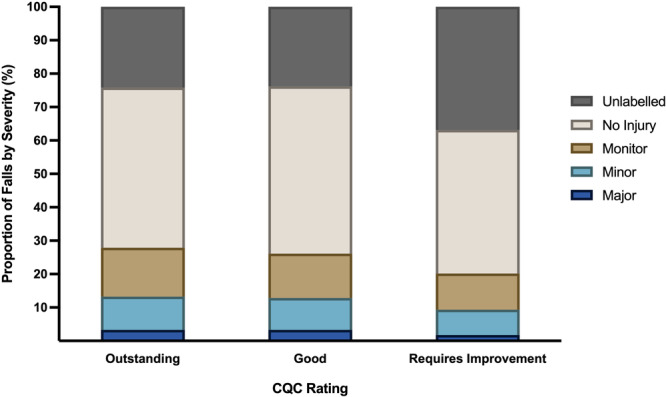
Distribution of falls by injury severity category and Care Quality Commission (CQC) rating. The CQC is the independent regulator of health and social care in England and defines the care home quality using a four‐tier scale: Outstanding, Good, Requires Improvement, or Inadequate. Data include an ‘unlabelled’ falls severity category where injury severity was not documented. The percentage of falls with ‘unlabelled’ falls severity is significantly higher in care homes that received a CQC rating of ‘requires improvement’ (*p* < 0.05).

Regarding regulatory oversight, care homes with an ‘inadequate’ rating were excluded due to insufficient sample size. Neither fall incidence rate nor hospitalisation incidence rate were related to CQC rating. Although fall injury severity was not related to CQC rating, falls with no documented fall injury severity were significantly more common in care homes with a CQC rating 'requires improvement' (Figure 4).

### 3.4 Logistic Regression

A univariable logistic regression demonstrated that 15 variables were statistically significantly associated with falling (Supporting Table S2). A multivariable analysis showed 9 of these variables were also independently associated with falling (Table 1). Variables associated with an increased risk of falling included increased frequency of exercise and toileting, whereas variables associated with a decreased risk of falling included higher and better nursing and handling risk profiles.

**Table 1:** Multivariable logistic regression analysis of routinely collected variables independently associated with residents living in care homes who experienced at least one fall (*N* = 12,012). The analysis includes 6,006 residents who experienced at least one fall (cases) and 6,006 matched residents with no documented falls (controls).

Variable	OR	95% CI for OR	*p*‐value
Exercise action frequency	8.08	4.01–16.28	< 0.001
Toilet action frequency	4.44	2.18–9.02	< 0.001
Mean Barthel index risk assessment score	4.37	1.26–15.20	0.02
Medical special observed frequency	3.04	1.53–6.03	0.001
Medical regular intervene frequency	2.96	1.36–6.45	0.006
Food action frequency	2.40	1.39–4.16	0.002
Respiration measurement frequency	0.24	0.08–0.71	0.01
Mean moving & handling risk assessment score	0.07	0.02–0.19	< 0.001
Mean amount of fluid (L/day)	0.06	0.03–0.11	< 0.001

*Note:* Data are presented as OR [95% CI]. Statistical significance was defined as *p* < 0.05.

Abbreviations: CI, confidence interval; L/day, litres per day; OR, odds ratio.

## 4 Discussion

### 4.1 Key Findings

This study utilised data collected from more than 1,700 care homes across England using the GDPR‐compliant MCM mobile app. Data were recorded continuously in real time using an icon‐driven interface including information on activities, food and fluid consumption, medication administration, exercise activities, and mental stimulation. The study focused on residents who were aged 75 years or over, not bed bound, with at least one full year of MCM app documentation.

The study identified 23,621 falls among 6,006 participants in 1 year. Fall incidence rate was positively associated with advancing age, and falls occurred at the highest rate during morning hours, and between February and June. The incidence of falls with major or minor injury was higher in males, and the majority of falls occurred indoors, most frequently in the bedroom.

A higher proportion of all‐cause hospitalisations was observed among residents who experienced at least one fall compared to those with no documented falls. Standard UK care pathways for falls involve an initial assessment by care staff and often require hospital transfer for acute assessment, particularly when a head injury or fracture is suspected. The hospitalisation incidence rate in our study was 13% higher in men, and there was a lower frequency of hospitalisations over the weekend. No significant association was shown between fall injury severity or hospitalisation rates, and CQC ratings.

We identified nine variables that were independently associated with the risk of falling. Notably, higher frequencies of recorded exercise and toileting were associated with an increased risk of falling, while higher fluid intake was associated with a lower risk of falling. These factors could be used as a foundation for building a fall‐risk assessment algorithm. It is possible that a higher frequency of exercise interventions was recorded for residents who remain ambulatory but exhibit postural instability. Similarly, increased toileting documentation may reflect acute changes in physiological status, such as altered bowel habits, indicative of clinical deterioration. While both scenarios are associated with a higher fall risk we could not extract this granular detail from the anonymised data provided.

### 4.2 Comparison With Established Risk Factors

Our findings reflect other studies, indicating that falls among residents living in care homes are common and exhibit similar patterns, including differences based on time of day and location. A cross‐sectional study in Nova Scotia demonstrated 56.2% of residents fell at least once over the 5 month study period [16]. A New York study found falls were most common between 4 pm and 8 pm, and least common during the night shift [17]. Most falls occurred in residents' bedrooms or bathrooms in a Bavarian prospective study at 528 care homes [18].

Many causes for falls in care homes have been published [19]. Common precipitating factors include gait and balance disorders, environmental hazards and visual impairment [20]. Other independent risk factors include male sex, dementia and certain medications [16]. A longitudinal study of 437 care homes found insomnia increased risk of falls with an adjusted OR of 1.52 [21]. One meta‐analysis found residents living in care homes were more than twice as likely to fall if there was a history of falls, walking aid use or moderate disability [22]. In our dataset a higher degree of mobility intervention appears to correlate with a higher fall risk.

In a study of 2,510 residents living in care homes, benzodiazepine use was associated with a 44% increase in the rate of falls [23]. A univariable analysis aggregating data from 16 studies showed muscle weakness conveyed the highest relative fall risk [24]. A Cochrane review study found that although Vitamin D reduced the falls rate, it did not reduce the overall fall risk [25].

Older people living in care homes are three times more likely to fall than those living in their own home [5] and are 10 times more likely to sustain serious injury [26]. One randomised controlled trial (RCT) compared 45 care homes using standard of care, with 39 care homes trialling the Guide to Action for falls prevention in Care Homes (GtACH) programme. The GtACH programme cohort had fewer falls and was considered cost effective when using the EuroQol‐5 dimensions‐based Quality Adjusted Life Years proxy with an incremental cost of £190.62 for every fall averted [27]. It is paramount to understand the patterns and risk factors associated with falls among residents living in care homes in order to implement effective cost‐efficient prevention strategies.

While the existing literature demonstrates well‐established falls risk factors, many previous studies are limited by recall bias and the static nature of periodic assessments. The novel contribution of our study lies in using continuous, real‐time data to capture the longitudinal clinical status of residents in care homes at an exceptional scale. This confirms that routinely recorded care activities show significant associations with falling that align with and build upon traditional geriatric assessments.

### 4.3 Algorithmic Fall Risk Prediction

Multiple fall risk assessment tools have been designed but few predictive fall risk models have been developed. In 2016, Marier et al published a repeated events survival model to analyse Minimum Data Set (MDS) 3.0 and Electronic Medical Records (EMR) data for 5129 residents living in 13 care homes within a single large California chain. They identified risk factors for falling but only a third of observed falls occurred among residents with the 10% highest risk profiles [28]. Ye et al used Electronic Health Record (EHR) data on patients over the age of 65 years to develop a machine‐learning‐based fall risk predictive tool, which successfully predicted over half the falls occurring within the first 60 days of the following year [29]. One Portuguese case‐control study across 25 care homes identified static balance as the best falls risk predictor achieving a receiver operating characteristic (ROC) curve accuracy of 71% [30].

The primary clinical utility of our approach is the demonstration that dynamic care variables are independently associated with the risk of falling. Unlike existing models that rely on static health records, our methodology utilises routinely captured digital care logs to identify behavioural trends. While these are currently characterised as independent associations, they provide the empirical foundation for real‐time fall risk prediction algorithms.

### 4.4 The Value of Detecting Fall Risks

Care home staff can be trained to manage falls effectively using available resources and training programmes such as the 'React to Falls' training resource [31]. A multicentre RCT comparing a multifactorial falls prevention programme with standard care in UK care homes for older adults showed the programme was cost effective with a reduced incidence rate ratio of 0.57 without a decrease in activity or increase in dependency [32]. A meta‐analysis found that interventions delivered with extra staffing and expertise led to reduced fall rates [33].

One initiative achieved a 36.6% reduction in falls rate through training and implementation of regular huddles for care home staff to improve information flow [26]. Pasquetti et al encourage staff to view falls in older people as a 'geriatric syndrome' and manage falls through multidimensional assessment with an integrated treatment and rehabilitation programme [34]. They advocate adopting the Comprehensive Geriatric Assessment (CGA) to evaluate and reduce fall risk in older persons. This includes assessment of the patient's functional capacity, need for support services and pharmacological burden. Treatment focuses on addressing inadequate lighting and enhancing residual capacity through neuromotor rehabilitation. Aggregated data such as ours, collected in real time, could shed light on specific local issues and compare them to general trends. This might facilitate highly targeted interventions.

Falls data capture in care homes traditionally relied on inconsistent paper‐based records. The digitisation of care records offers an opportunity to overcome this barrier by enabling consistent large‐scale data collection. Our study demonstrates the feasibility of capturing such data, aligning with UK priorities such as the Developing resources And minimum data set for Care Homes' Adoption (DACHA) and Minimum Operational Data Standard (MODS) to create scalable and interoperable data infrastructures [35, 36]. This methodological advance provides an opportunity for robust research in an area where high‐quality data have historically been lacking. Consequently, health systems should incentivise the transition to real‐time digital recording.

### 4.5 Limitations

To our knowledge this is the largest study utilising continuous data collection from an icon‐driven mobile app. The wide range of data collected, including physical and mental wellbeing, provided a holistic and nuanced analysis of factors associated with fall risk, while avoiding recall bias through real‐time data entry. However, several limitations remain.

#### 4.5.1 Data and Documentation

The quality of data entered depends on the accuracy of user input. Many subjective assessments result in recording inconsistencies between staff, although in a dataset this large, these would appear as noise, so remaining correlations are likely to be real. Real‐time data may lead to documentation inaccuracies due to time pressures faced by staff. Furthermore, because the app records all‐cause hospitalisation and the anonymisation protocol precluded external data linkage, it was not possible to differentiate between admission directly resulting from falls and those due to unrelated medical conditions.

#### 4.5.2 Selection Bias and Study Design

Selection bias may have been introduced by the requirement for one full year of documentation in care homes using digital monitoring, potentially favouring higher‐resourced settings and limiting generalisability. Despite adjusting for multiple variables, residual confounding is likely as routinely collected data did not include factors such as medication use. Additionally, the study is susceptible to temporal ambiguity. Aggregating data over the 6‐month period preceding a fall allowed for the identification of stable behavioural patterns; however, it is possible that some associations, such as increased care frequency, represent a staff response to a resident's functional decline rather than a primary risk factor for the subsequent fall.

#### 4.5.3 Statistical Considerations

There are several statistical considerations. First, as the analysis did not explicitly account for care home clustering, care home‐specific practices may have influenced associations. However, as the data were taken from more than 1,700 care homes, the results represent a broad cross‐section of institutions reducing the likelihood that the results are driven by unique practices from a small number of care homes. Second, assessments for multicollinearity were not performed and correlations between routinely recorded care activities may exist, which can affect the precision and estimated weight of the individual odds ratios. Third, the very low odds ratio for fluid intake (0.06) is influenced by the 1‐litre scaling unit used in the model. While the association is statistically significant, the magnitude of this effect size appears large given 1 litre is a substantial volume of fluid for this study cohort. Finally, as model diagnostics were not performed, these results should be interpreted as independent associations providing a foundation for future research, rather than as a validated tool for individual fall risk prediction.

## 5 Conclusion

This study provides valuable insights into the patterns and risk factors related to falls among residents living in care homes through a comprehensive analysis of real‐time digital data collection via the MCM mobile app. Our findings identify independent associations and offer a methodological and empirical foundation for the future development of prospective fall‐risk assessment models. Accurately identifying residents living in care homes at high risk of falling, and instigating data‐driven falls prevention programmes, may help reduce falls.

## Author Contributions

**Julian Gertner:** writing – original draft, interpretation, formal analysis. **Ofir Dvir:** writing – original draft, formal analysis. **Niv Shifrin:** formal analysis. **Paul Wolfson:** writing – original draft. **Robert Moskovitch:** writing – review and editing. **Laurence B. Lovat:** conceptualisation, methodology, data curation, interpretation, supervision, writing – review and editing.

## Conflicts of Interest

Julian Gertner, Ofir Dvir, Niv Shifrin, Paul Wolfson, Robert Moskovitch, Laurence B. Lovat: No conflicts to disclose.

## Transparency Statement

The lead author Julian Gertner affirms that this manuscript is an honest, accurate, and transparent account of the study being reported; that no important aspects of the study have been omitted; and that any discrepancies from the study as planned (and, if relevant, registered) have been explained.

## Supporting information

Supporting File

## Data Availability

The data that support the findings of this study were provided by a third party (Person Centred Software, Guildford, UK) and are subject to a data‐sharing agreement that precludes public sharing. Inquiries regarding data access should be directed to the third‐party provider via their website (https://personcentredsoftware.com) or email (hello@personcentredsoftware. com).
