# Supplementary material for: Falls Among Residents Living in Care Homes Using Real‐Time Data Collection: A Large UK Case‐Control Study
Source: Health Sci Rep. 2026 Apr 19;9(4):e72350. doi: 10.1002/hsr2.72350 (PMC13092222; doi:10.1002/hsr2.72350)
Supplement: Supplementary file 1 — Supporting File [file HSR2-9-e72350-s001.docx]

Supplementary Table S1: Definitions for routinely collected variables that staff recorded using the MCM app for residents living in care homes.

| Variable | Definition | Examples/Additional notes |
| --- | --- | --- |
| 1. Exercise Action Frequency | Total number of exercise-related activities performed by residents | Indoor skittles, walking, swimming, horse riding, bowling, ‘Oomph!’ exercises |
| 2. Mean Barthel Index Risk Assessment Score | Average score derived from intermittent Barthel Index assessments which evaluates residents’ ability to perform basic activities of daily living | Mobility, self-care, continence |
| 3. Food Action Frequency | Total number of eating-related events | Meals (breakfast, lunch, teatime), snack, food orders, eat out, soup |
| 4. Toilet Action Frequency | Total number of toileting-related activities | Bowel movements, urination, assistance with toileting, soiled clothes, pad or catheter change |
| 5. Fluid Action Frequency | Total number of fluid intake-related events | Water, juice, hot drinks, coffee, wine, thickened fluids |
| 6. Weight Measurement | Recorded body weight (kg) of residents |  |
| 7. Mean Amount of Fluid | Average estimated daily fluid intake (L) recorded for each resident | Derived from proportions of cups of fluid consumed |
| 8. Mean Dependency Risk Assessment Score* | Average score derived from intermittent assessments of resident’s level of dependency in performing daily activities | Daily activities included: bathing, continence, dressing, mobility |
| 9. Mean Moving & Handling Risk Assessment Score* | Average score derived from intermittent moving and handling risk assessments | Risk factors included: level of assistance and equipment required for transfers or toileting |
| 10. Medical Regular Intervene Frequency | Total number of routine medical interventions | Medication administration, capillary blood glucose monitoring |
| 11. Respiration Measurement Frequency | Total number of times respiration measurements were recorded for residents | Measurements included: Respiration rate |
| 12. Mean Fall Risk Assessment Score* | Average score assessing risk of falls for each resident |  |
| 13. Mean Nutritional Risk Assessment Score* | Average score evaluating nutritional status and risk of malnutrition |  |
| 14. Medical Special Observed Frequency | Total number of specialised medical observations | Oxygen flow rate, pain scores, calling a doctor or ambulance |
| 15. Medical Uncertain Intervene Frequency | Total number of medical interventions requiring specialist input |  |
| 16. Illness Action Frequency | Total number of illness-related events | Attending to a resident for feeling unwell |
| 17. Mean Waterlow Risk Assessment Score | Average score derived from intermittent Waterlow Score1 assessments which evaluates risk of developing pressure ulcers |  |
| 18. Nutrition watch Action Frequency | Total number of times nutritional status or activity was monitored | Taking oral supplements, meal consumption |
| 19. Blood O2 Measurement | Average (mean) blood oxygen saturation (%) level recorded for residents |  |
| 20. Medical Special Intervene Frequency | Total number of special nursing interventions | Wound care, catheter changes, enteral tube management, stoma care, physiotherapy |
| 21. Mobility assistance Action Frequency | Total number of times mobility assistance was provided to residents | Walking aids, physical support |
| 22. Mean Eating Drinking Risk Assessment Score* | Average score derived from intermittent assessments of risks related to eating and drinking | Choking, poor appetite |
| 23. Medical Regular Observed Frequency | Total number of medical observations | Blood pressure, pulse, temperature, oxygen saturation, skin integrity |
| 24. Emotions Action Frequency | Total number of emotional or behavioural observations | Anger, confusion, repetitive behaviours, emotional support provided to residents |
| 25. Mental Capacity Risk Assessment Frequency | Total number of times mental capacity assessments were conducted on each resident |  |
| 26. Mental Capacity Risk Assessment Score | Average score from intermittent mental capacity assessments evaluating cognitive and decision-making ability | Using Mental Capacity Act (MCA)2 framework |
| 27. Blood Sugar Measurement | Average (mean) capillary blood sugar level (mmol/L) recorded for residents |  |
| 28. Fluid Watch Action Frequency | Total number of times that events related to fluid intake or hydration status were recorded | Events included: drinking glass of juice |
| 29. Height Measurement Frequency | Total number of times a resident’s height was recorded |  |
| 30. Entertainment Action Frequency | Total number of times residents participated in entertainment-related activities | Concerts, TV, arts and crafts, pet therapy, quizzes, games |
| 31. Mean Appetite Score* | Average score derived from intermittent assessments of a resident’s appetite level |  |
| 32. Mean Happiness Score* | Average score derived from intermittent assessments of a resident’s happiness | Likert scale with Happiness slider comprising of faces (e.g. smiley, sad) used to represent residents’ emotions |
| 33. Diet Action Frequency | Total number of dietary-related adjustments | Special meal plans, food supplements, prescribed dietary changes |
| 34. Smoking Action Frequency | Total number of times a resident smokes |  |

*specific scoring system not revealed to the study team; a standardised method was consistently applied across all residents

Supplementary Table S2: Univariate logistic regression analyses of routinely collected variables associated with residents living in care homes who experienced at least one fall (*N*=12,012). The analyses include 6,006 residents who experienced at least one fall (cases) and 6,006 matched residents with no documented falls (controls).

| Variable | *OR* | *95% CI for OR* | *P-value* |
| --- | --- | --- | --- |
| Exercise Action Frequency | 12.62 | 7.05-22.57 | <.001 |
| Mean Barthel Index Risk Assessment Score | 9.98 | 3.22-30.98 | <.001 |
| Food Action Frequency | 5.98 | 3.49-10.23 | <.001 |
| Toilet Action Frequency | 4.37 | 2.71-7.05 | <.001 |
| Fluid Action Frequency | 3.08 | 2.15-4.41 | <.001 |
| Weight Measurement | 0.48 | 0.32-0.72 | <.001 |
| Mean Amount of Fluid (L/day) | 0.15 | 0.09-0.27 | <.001 |
| Mean Dependency Risk Assessment Score | 0.28 | 0.12-0.61 | .001 |
| Mean Moving & Handling Risk Assessment Score | 0.20 | 0.08-0.51 | .001 |
| Medical Regular Intervene Frequency | 2.35 | 1.36-4.07 | .002 |
| Respiration Measurement Frequency | 4.11 | 1.61-10.48 | .003 |
| Mean Fall Risk Assessment Score | 0.38 | 0.19-0.74 | .005 |
| Mean Nutritional Risk Assessment Score | 2.28 | 1.18-4.39 | .01 |
| Medical Special Observed Frequency | 0.35 | 0.14-0.84 | .02 |
| Medical Uncertain Intervene Frequency | 5.88 | 1.27-27.2 | .02 |
| Illness Action Frequency | 1.76 | 0.99-3.14 | .05 |
| Mean Waterlow Risk Assessment Score | 0.43 | 0.18-1.02 | .05 |
| Nutrition watch Action Frequency | 2.37 | 0.98-5.74 | .05 |
| Blood O2 Measurement | 0.32 | 0.08-1.25 | .11 |
| Medical Special Intervene Frequency | 3.16 | 0.75-13.31 | .12 |
| Mobility assistance Action Frequency | 0.72 | 0.45-1.15 | .17 |
| Mean Eating Drinking Risk Assessment Score | 1.86 | 0.72-4.82 | .20 |
| Medical Regular Observed Frequency | 0.57 | 0.2-1.59 | .28 |
| Emotions Action Frequency | 4.20 | 0.28-62.65 | .30 |
| Mental Capacity Risk Assessment Frequency | 3.29 | 0.26-41.23 | .36 |
| Mental Capacity Risk Assessment Score | 1.17 | 0.81-1.70 | .41 |
| Blood Sugar Measurement | 0.15 | 0.00-29.43 | .48 |
| Fluid Watch Action Frequency | 1.36 | 0.56-3.35 | .50 |
| Height Measurement Frequency | 0.85 | 0.52-1.38 | .50 |
| Entertainment Action Frequency | 0.73 | 0.25-2.13 | .56 |
| Mean Appetite Score | 1.07 | 0.52-2.17 | .86 |
| Mean Happiness Score | 0.92 | 0.27-3.06 | .87 |
| Diet Action Frequency | 0.94 | 0.36-2.46 | .90 |
| Smoking Action Frequency | 0.95 | 0.10-14.20 | .98 |

*Abbreviations: OR, odds ratio; CI, confidence interval; L/day, litres per day.*

*Note: Data are presented as OR [95% CI]. Statistical significance was defined as p<.05.*

Supplementary References:

1. Waterlow J. Pressure sores: a risk assessment card. *Nurs Times*. 1985;81(48):49-55.

2. *Mental Capacity Act 2005, c. 9. Available at: Https://Www.Legislation.Gov.Uk/Ukpga/2005/9/Contents/Enacted (Accessed: 28 January 2025)*. Accessed January 28, 2025. https://www.legislation.gov.uk/ukpga/2005/9/contents/enacted

**Supplementary Table S3:** Classification of fall injury severity and binary grouping for analysis. Fall injury severity was judged by staff at the point of care and categorised using four available levels: major, minor, monitor, and no injury. These were subsequently grouped into a binary classification: falls with injury (major or minor injuries) and falls without injury (monitor or no injury).

| Recorded Severity Category | Criteria | Binary Grouping |
| --- | --- | --- |
| Major | Point-of-care staff judgement | Fall with injury |
| Minor | Point-of-care staff judgement | Fall with injury |
| Monitor | Point-of-care staff judgement | Fall without injury |
| No injury | Point-of-care staff judgement | Fall without injury |
